# *AidP*, a novel *N*-Acyl homoserine lactonase gene from Antarctic *Planococcus* sp.

**DOI:** 10.1038/srep42968

**Published:** 2017-02-22

**Authors:** Wah Seng See-Too, Robson Ee, Yan-Lue Lim, Peter Convey, David A. Pearce, Wai-Fong Yin, Kok-Gan Chan

**Affiliations:** 1 Division of Genetics and Molecular Biology, Institute of Biological Sciences, Faculty of Science University of Malaya, 50603 Kuala Lumpur, Malaysia; 2 National Antarctic Research Centre (NARC), Institute of Postgraduate Studies, University of Malaya, 50603 Kuala Lumpur, Malaysia; 3 British Antarctic Survey, NERC, High Cross, Madingley Road, Cambridge CB3 OET, UK; 4 Faculty of Health and Life Sciences, University of Northumbria, Newcastle Upon Tyne NE1 8ST, UK; 5 UM Omics Centre, University of Malaya, Kuala Lumpur, Malaysia

## Abstract

*Planococcus* is a Gram-positive halotolerant bacterial genus in the phylum Firmicutes, commonly found in various habitats in Antarctica. Quorum quenching (QQ) is the disruption of bacterial cell-to-cell communication (known as quorum sensing), which has previously been described in mesophilic bacteria. This study demonstrated the QQ activity of a psychrotolerant strain, *Planococcus versutus* strain L10.15^T^, isolated from a soil sample obtained near an elephant seal wallow in Antarctica. Whole genome analysis of this bacterial strain revealed the presence of an *N*-acyl homoserine lactonase, an enzyme that hydrolyzes the ester bond of the homoserine lactone of *N*-acyl homoserine lactone (AHLs). Heterologous gene expression in *E. coli* confirmed its functions for hydrolysis of AHLs, and the gene was designated as *aidP* (autoinducer degrading gene from **P*lanococcus* sp.). The low temperature activity of this enzyme suggested that it is a novel and uncharacterized class of AHL lactonase. This study is the first report on QQ activity of bacteria isolated from the polar regions.

Quorum sensing (QS) or bacterial cell-to-cell communication has become a focus of research due to its high potential as a novel application to prevent the onset of bacterial infections and reduce the current over-use of antibiotics which itself is a selective pressure leading to increased antibiotic resistance^1^. Bacteria communicate with each other to control numerous phenotypic characteristics, such as the production of virulence factors^2^, antibiotic biosynthesis^3^, and biofilm differentiation^4^. In nature, QS could be highly advantageous particularly in the contexts of symbioses and niche adaptation, and for facilitating population migration towards/away from favourable/unfavourable conditions in their local environment^5^. Antarctica provides some of the most challenging environments on Earth for life^6^,^7^. Metagenomic analysis of Antarctic soil has revealed that Antarctic microbial communities are more complex and higher diversity than previously thought^8^. The presence of QS genes in Antarctic soil, together with antibiotic, biofilm formation, virulence and other toxic compound resistance genes, suggests that QS provides these bacteria with a competitive advantage in hostile Antarctic environments^9^.

The disruption of QS signals, termed quorum quenching (QQ), was first described by Dong *et al*.^10^ in *Bacillus* sp. However, QQ activity in extremophiles is not well studied, and has only been characterized in detail in a thermophile, *Geobacillus kaustophilus*^11^. To our knowledge, there is no report or characterization of QQ activity in bacteria isolated from the polar regions. With the ability to disrupt intercellular communication, QQ potentially makes an important contribution to competitive ability.

*Planococcus* is a member of the family *Planococcaceae*, a group of halotolerant Gram-positive bacteria^12^ commonly found in various habitats in Antarctica^13^,^14^. In this study, we report QQ activity in Antarctic *Planococcus versutus* strain L10.15^T^, that was capable of inactivating synthetic *N*-acyl homoserine lactones (AHLs) with acyl side chain lengths C_4_-C_12_, and functioning at a temperature as low as 4 °C. The gene responsible for the QQ activity of *P. versutus* L10.15^T^ was identified and confirmed for its function in an expression study. The cold-active characteristic of the enzyme coded by this gene suggested that it belonged to a novel class of *N-*acyl homoserine lactonase, and we therefore term the gene as ‘autoinducer degrading gene from *Planococcus* sp.’ (*aidP*). The discovery of the AidP protein provides the foundation for new routes of prospecting QQ enzymes for industrial applications.

## Results

### Isolation and molecular identification of QQ bacteria

The enrichment KG medium became turbid after one week of incubation, suggesting that the bacteria were able to utilise the synthetic AHL (C_6_-HSL) as sole carbon source^15^. The bacteria were then screened for QQ activity in an AHL inactivation assay using the CV026 biosensor. Amongst the 15 bacterial isolates obtained, strain L10.15^T^ demonstrated strong QQ activity and was selected for further analysis. Web-based similarity comparison against the GenBank database suggested that strain L10.15^T^ belonged to the genus *Planococcus*, and this was confirmed by phylogenetic analysis. Phylogenetic analysis of 16S rRNA gene sequences indicated that the strain’s closest relatives were *P. halocryophilus, P. kocurii* and *P. donghaensis.* Polyphasic taxonomic study confirmed that strain L10.15 represents a new taxon within *Planococcus*, which we designated as *P. versutus* L10.15^T ^16.

### Degradation of AHLs

QQ activity of *P. versutus* L10.15^T^ was verified using synthetic AHL (C_6_-HSL) screened with biosensor CV026. Synthetic C_6_-HSL was selected for initial screening since it was used as the sole source of carbon in the enrichment medium. Strain L10.15^T^ cells degraded 100 μM of C_6_-HSL in 100 μl of cell suspension within 24 h (Supplementary Fig. S1). As AHLs will undergo lactonolysis under alkaline conditions^17^, turnover of AHLs by alkaline lactonolysis was ruled out as no change in pH values was observed in the reaction mixtures after 24 h (data not shown).

**Figure 1:**
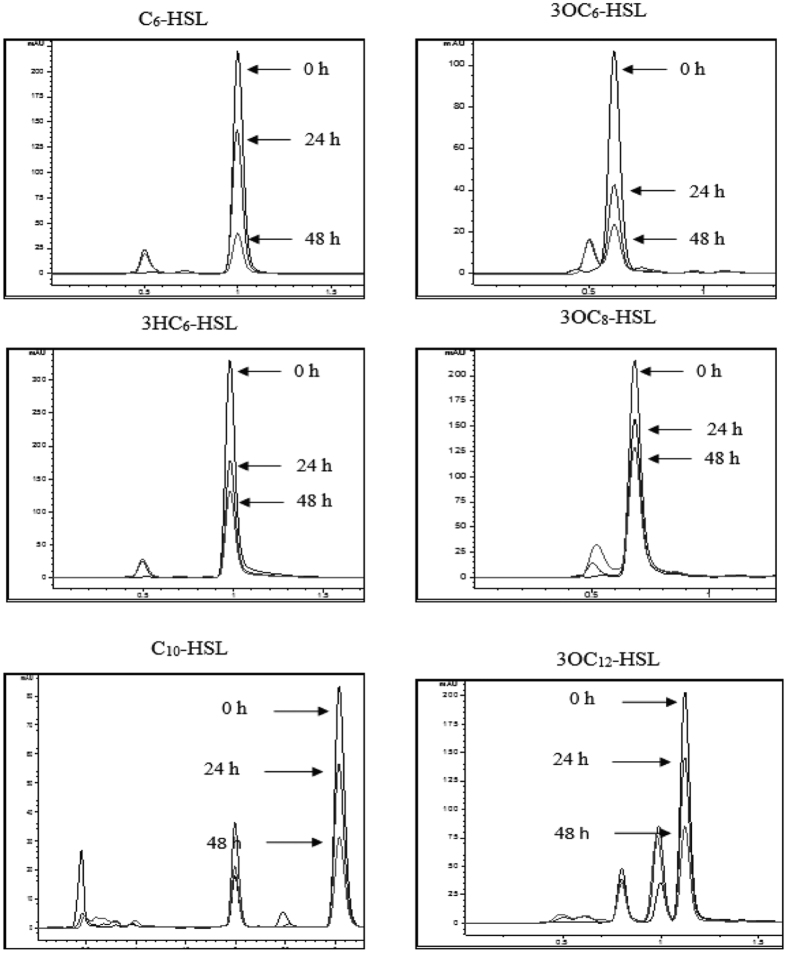
Analysis of *P. versutus* L10.15^T^ AHL degradation by RRLC.

Because biosensor CV026 is only applicable for detection of short chain AHLs, rapid resolution liquid chromatography (RRLC) (Fig. 1) was used to confirm the QQ activity of strain L10.15^T^. A range of AHLs (C_4_-C_13_-HSL, with or without substituted groups) was tested. The results indicated that *P. versutus* L10.15^T^ degraded all the AHLs tested and exhibited high activities toward most, including those with 3-hydroxy or 3-oxo substitutions and un-substituted homoserine lactones. The substrate specificity was significantly affected by the length of the acyl acid chain, with the estimated relative activity of strain L10.15^T^ showing a gradual decease toward AHLs with longer chain lengths. The strain had low QQ activity toward C_13_-HSL (data not shown). No AHL degradation was observed in all control AHL degradation assays repeated with *E. coli* DH5α cells and PBS.

### Determination of AHL-lactonase activity *via* acidification assay

In order to demonstrate that *P. versutus* L10.15^T^ produced an AHL-degrading enzyme, the bacterial suspension was boiled at 95 °C for 30 min to denature any enzyme present before addition to the synthetic AHLs. The boiled cell suspension no longer possessed QQ activity (data not shown), indicating that the activity was most probably catalyzed by an enzyme. An acidification assay^17^ was conducted to re-lactonise the opened ring of AHL lactones caused by the AHL-lactonase. A proportion of the AHL amounts degraded by strain L10.15^T^ was restored after acidification, with C_4_-HSL being restored up to 30% (Supplementary Fig. S2), further supporting that the QQ activity of *P. versutus* L10.15^T^ may be due to an AHL-lactonase.

### Complete genome sequencing and assembly of strain L10.15

Sequenced data of *P. versutus* L10.15^T^ comprised a total of 135,526 polymerase reads (Reads N50: 9333 bp) which, upon filtering, resulted in a total of 167,910 subreads (Reads N50: 7768 bp). The subreads were subjected to *de novo* assembly using Hierarchical genome assembly process (HGAP) algorithm version 2, which constructed a final assembly (mean coverage: 219.3 times) of three circular contigs with sizes of 3.2 Mb, 70.7 kb and 9.8 kb, respectively. All contigs contained self-overlapping ends, indicating their circularity. The coordinates of the overlapping ends were estimated using the gepard dotplot tool and were subsequently trimmed to generate blunt-ended circular contigs.

BLASTn searches against the NCBI non-redundant nucleotide database provided a preliminary identity assignment for each contig, with the largest contig being determined as a chromosomal contig and the two smaller contigs as being extrachromosomal contigs, and most likely representing plasmids due to sequence similarity to deposited plasmid sequences (Supplementary Tables S1–S3). Based on these results, designations of pPS15-1 and pPS15-2 were given to the 70.7 kb and 9.8 kb contigs, respectively.

Further support for the identity of pPS15-1 and pPS15-2 was provided by the identification of genes associated with the replication module of plasmids. For example, in pPS15-1, a putative *parA* (WP_007723335.1), and in its opposite transcriptional orientation, 2 CDSs (WP_049694985.1 and WP_049694987.1) were identified, genes which encode putative proteins with sequence similarities to various plasmid replication proteins. In pPS15-2 a putative RepB family plasmid replication initiator protein gene (WP_049694148.1), a common occurrence among the plasmids of cold-active bacteria, was identified^18^.

The chromosomal origin of the 3.2 Mb contig was further established through the identification of a 9.8 kb section which contains a 708 bp putative *oriC* region (9 DnaA boxes) (Supplementary Figure S3) flanked by a gene cluster which comprised *rmpH* (WP_049693545.1), *rnpA* (WP_049693546.1), *dnaA* (WP_049693544.1), *dnaN* (WP_049693543.1), *yaaA* (WP_049693542.1), *recF* (WP_049693541.1), *gyrB* (WP_049693540.1), and *gyrA* (WP_049693539.1). The chromosome was rearranged to begin with the putative *dnaA* CDS (1 to 1344 nt) and to end with the putative *oriC* region (3248918 to 3249625 nt). In order to obtain an *in silico* evaluation of the degree of completeness of the chromosome, a Z-curve was then plotted using the rearranged chromosome. From the plot, an inverted V-shape was observed for both AT- and GC- disparity curves, where the maximum (estimated location of putative replication terminus (*terC*) site) and the minimum (corresponding with the location of predicted *oriC* site) were situated at diametrically opposite locations (Supplementary Fig. S3). The presence of both the origin and terminus of replication provided an indication of the completeness of the chromosome.

### Overview of general genome features of strain L10.15^T^

The assembled genome of strain L10.15^T^ comprised three circular replicons with a mol% G + C content of 39.4%, which is consistent with the reported GC contents of *P. donghaensis* and *P. kocurii*^19^,^20^. A total of 3153 coding sequences (CDS), 9 rRNA operons and 71 tRNAs were predicted to be encoded by the genome (Table 1). The NCBI genome accession number for the chromosome is CP016540, for pPS15-1 is CP016541.2 and for pPS15-2 is CP016542.2.

### Identification of *Planococcus versutus* L10.15^T^ AHL-lactonase gene

Analysis of the annotated genome from the RAST server revealed the presence of a candidate QQ gene (annotated as an *N-*acyl homoserine lactonase). The 282 amino acid sequence was subsequently subjected to BLASTp search against the NCBI non-redundant protein sequences database. The comparison result revealed that the predicted proteome of the CDS showed this protein was annotated as an MBL fold metallo-hydrolase. BLASTp search also revealed a MBL fold metallo-hydrolases with 98% identity in *P. antarticus, P. faecalis* and *P. kocurii*, indicating that the gene is conserved across several *Planococcus* species. BLASTp search also indicated that genes with lower identity (<85%) were detected in *Bacillus, Jeotgalibacillus, Panaenibacillus, Lysinibacillus* and other genera from the Order Bacillales. However, only one AHL lactonase gene^21^ (from *Lysinibacillus* sp. Gs50) with identity 81% has been characterized. The BLASTp also detected several putative conserved domains (Supplementary Fig. S4) including AHL lactonase MBL-fold (Table 2).

**Figure 2:**
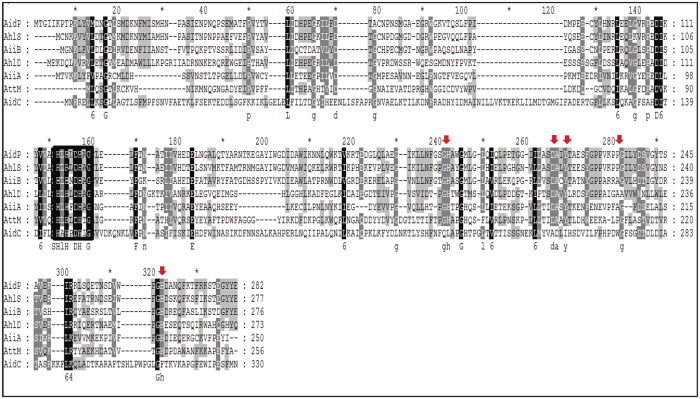
Comparison of amino acid sequences of *P. versutus* L10.15^T^ AidP and five known AiiA-type lactonases. The amino acid sequence of AidP (Genbank accession number WP_049694637.1) was compared with those AiiA from *Bacillus* sp.240B1 (accession number AAF62398), AiiB from *Agrobacterium tumefaciens* C58 (accession number AAK91031), AhlD from *Arthrobacter* sp. strain IBN110 (accession number AAP57766.1), AttM from *A. tumefaciens* strain A6 (accession number AAD43990), AidC from *Chryseobacterium* sp. strain StRB126 (accession number BAM 28988.1), and QlcA from the soil metagenome (accession number ABV58973.1). Sequences were aligned using the ClustalW program and shaded using the Genedoc program (http://www.nrbsc.org/gfx/genedoc/). The zinc-binding motif is boxed with rectangles. The amino acid residues that are essential for enzymatic activity of the known AHL lactonase are indicated by red arrows.

When the amino acid sequence of *aidP* was compared with those of the known AiiA-type lactonases (Fig. 2), the zinc-binding motifs HXHXDH~H, which are commonly conserved sequences of the metallo-β-lactamase superfamily, were found in both the known AiiA-type lactonases and in *aidP* (^117^HLHLDH^122^ ~ H^219^). The multiple alignment analysis of *aidP* and other AiiA-type lactonases also indicates (1) the active-site residue of the AHL lactonase enzyme, Tyr222, Leu120, Asp121, which are known to be conversed in most of the AiiA-type lactonases; (2) the amino acid residue that interacts with the primary ligand, H265 and D219; and (3) a non-conservative residue in the *N-*binding region, G235, was also detected. The catalytic mechanism involved in the active-site residue, and also the possible product-bound mechanism, are considered below.

**Figure 3:**
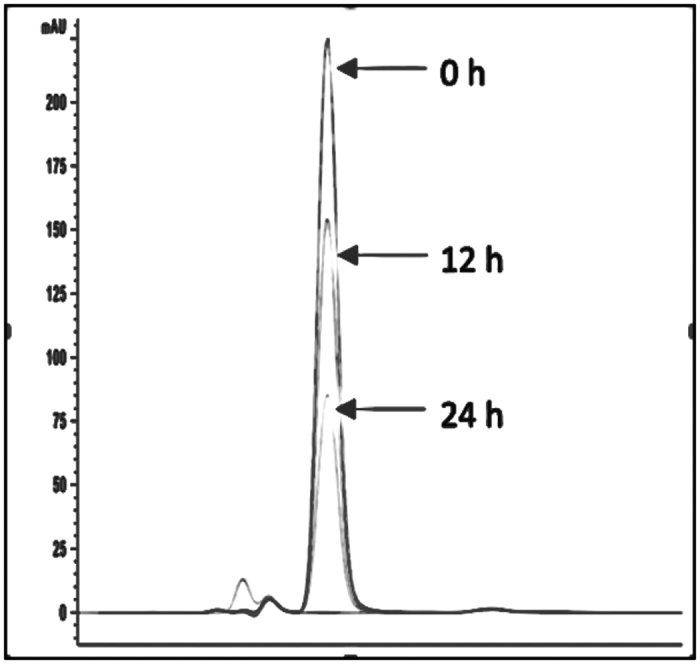
Heterologous expression study of *P. versutus* L10.15^T^ *aidP* in *E.coli* BL21 Star^TM^. The QQ inactivation assay was conducted at 16 °C for 24 h and subjected to RRLC analysis.

**Figure 4:**
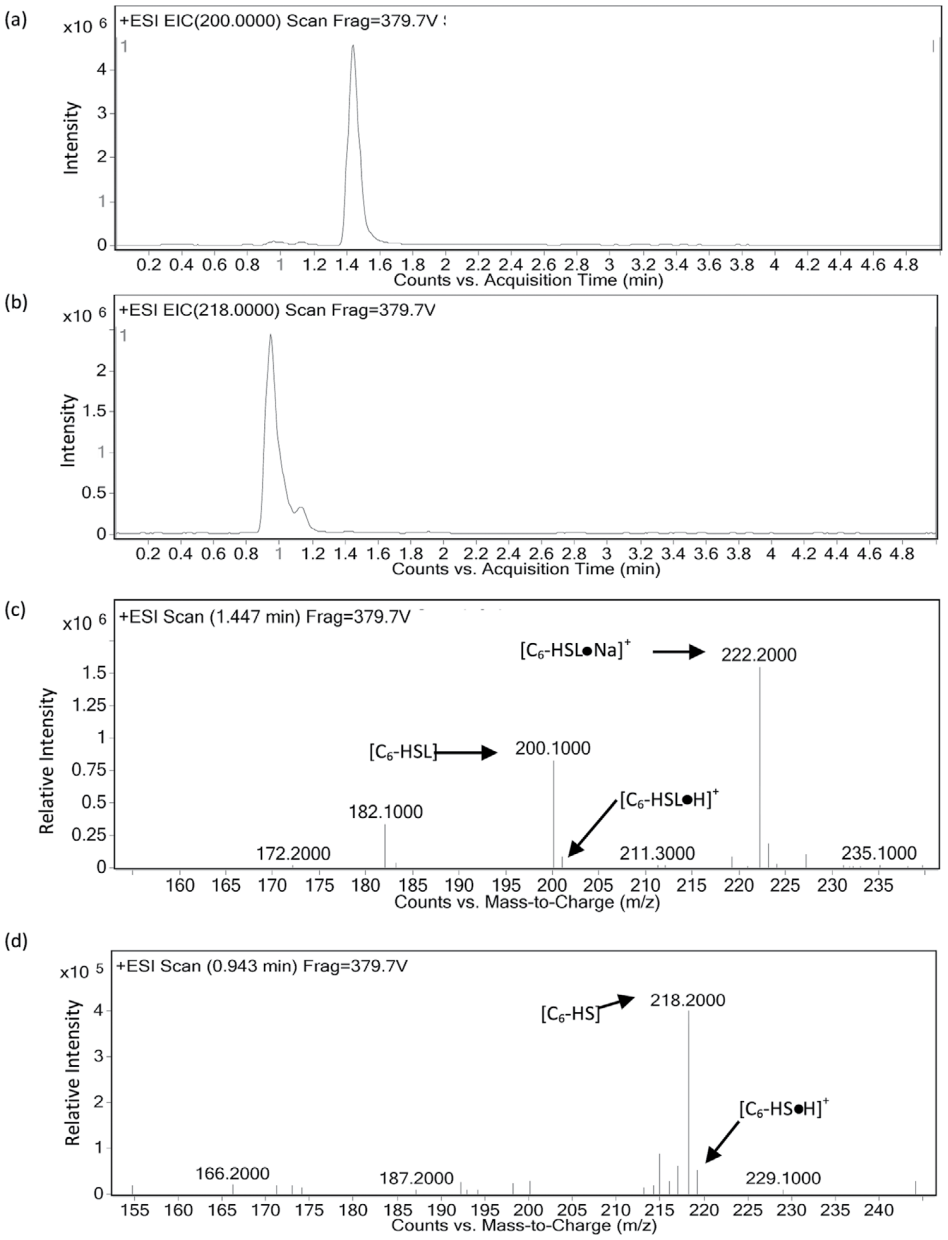
LC-MS analysis of the *P. versutus* L10.15^T^ *aidP* in *E. coli* BL21 Star^TM^ hydrolyzed C_6_-HSL product. (**a**) LC profile C_6_-HSL. (**b**) LC profile of hydrolysed C_6_-HSL (**c**) ESI-MS analysis of LC fractions containing the 1.48 min of undigested product (**d**) and 0.94 min of digested C_6_-HSL.

To confirm whether this candidate gene is responsible for the QQ activity in *P. versutus* L10.15^T^, it was ligated with expression vector pET-100 and cloned into *E. coli* BL21 Star^TM^. Recombinant *E. coli* BL21 Star^TM^ cells harboring the plasmids were screened for QQ activity with 10 μM C_6_-HSL using RRLC as described before. *E. coli* BL21 Star^TM^ containing the plasmid ligated with the candidate gene showed significant AHL-degrading activity against C_6_-HSL (Fig. 3). To further confirm the AHL degradation gene is an AHL lactonase gene, the AHL residue was analyzed by liquid chromatography-mass spectrometry (LC-MS). This analysis indicates retention time of C_6-_HSL was shifted from 1.45 min (Fig. 4a) to 0.94 min (Fig. 4b). ESI-MS analysis of C_6-_HSL with retention time 1.45 min exhibits a strong quasimolecule (M-H) ion at mass-to-charge ratio (*m/z*) of 200.0 (Fig. 4c). However, ESI-MS analysis of the AHL residue revealed a product with *m/z* of 218.0 (Fig. 4d). This result indicates that the C_6_-HSL experiences a mass increase of 18, corresponding to a water molecule, further suggesting that the candidate gene produced an enzyme that hydrolyzes the ester bond of the homoserine lactone ring of C_6_-HSL. This QQ gene, which may represent a novel class of AHL lactonase, was termed *aidP* (autoinducer degrading gene from *Planococcus* sp.).

### AidP is a new member of the AiiA-type AHL-lactonase

**Figure 5:**
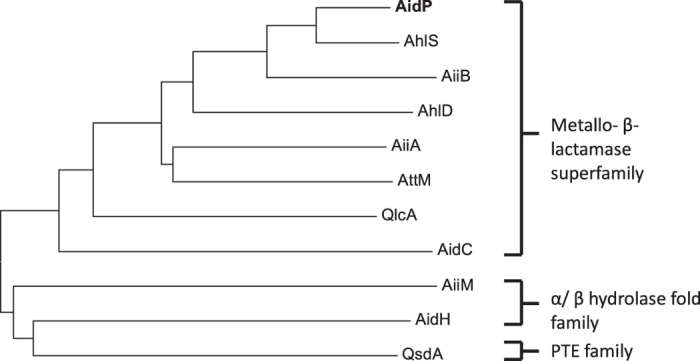
Phylogenetic analysis of *P. versutus* L10.15^T^ AidP. Phylogenetic tree based on amino acid sequences of AidP, AiiB, AhlD, AiiA, AttM, AidC, QlcA, AidH (accession number ACZ73823.1), and AidH from *Ochrobactrum* sp. strain T63, AiiM from *Microbacterium testaceum* strain StLB037 (accession number BAH97082.2), and QsdA from *Rhodococcus erythropolis*strain W2 (accession number AAT06802.1). The dendrogram was constructed by the neighbour-joining method using MEGA 6 software. The scale bar represents 0.2 substitutions per amino acid position.

**Figure 6:**
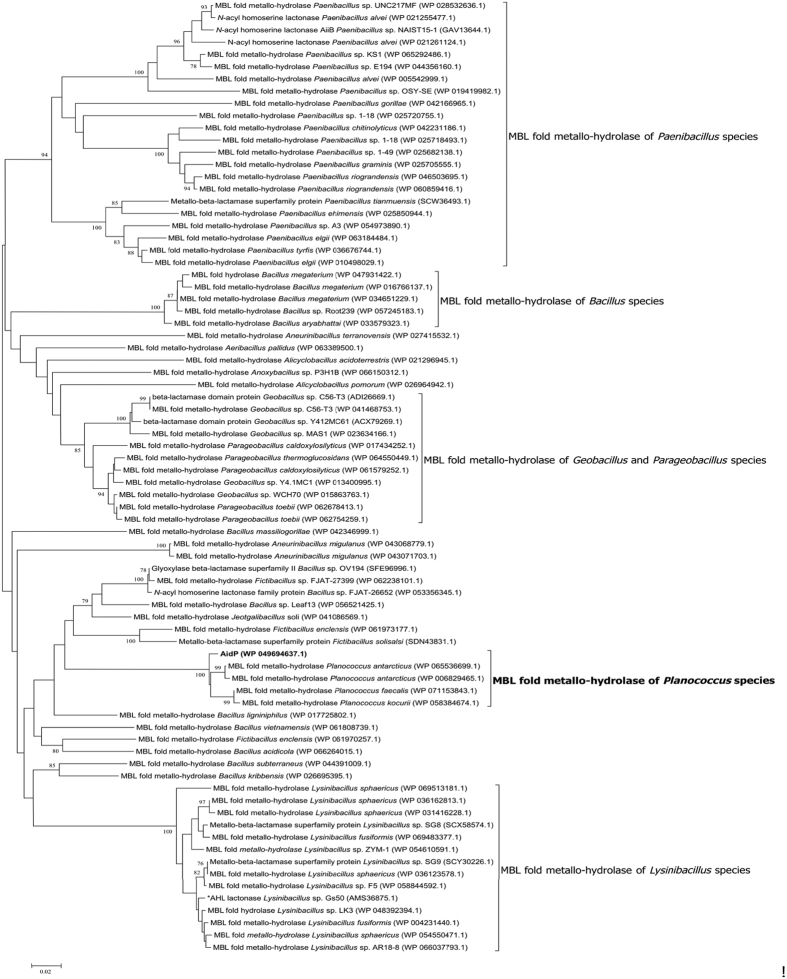
Neighbour-joining phylogenetic analysis of *P. versutus* L10.15^T^ *aidP* and other MBL fold metallo-hydrolases. Bootstrap values are expressed as percentages of 1000 replications. Bar, 0.02 substitutions per amino acid position.

To evaluate the novelty of AidP in the AHL-lactonase family, we used the amino acid sequence of *aidP* to determine the phylogenetic relationship between AidP and known AHL lactonases from various bacteria using the neighbour-joining method (Fig. 5). The AHL lactonase can be grouped into the metallo-β-lactamase superfamily, α/β-hydrolase-fold family, and the phosphotriesterase (PTE) family. Among these groups, the metallo-β-lactamase superfamily was well characterized from various bacteria. The amino acid sequence of *aidP* also clustered into the metallo-β-lactamase superfamily. However, it clustered distantly from the other lactonase clusters represented by six other AiiA-type lactonases. The *aidP* gene has low similarity to each of the known AiiA-type AHL lactonases - 26.2% identity across the entire length of AiiA from *Bacillus* sp. 240B1^22^, 24.9% identity across AttM from *Agrobacterium tumefaciens* strain C58^23^, 27.5% across AhlD from *Arthrobacter* sp. strain IBN110^24^, 29% identity across AidC from *Chryseobacterium* sp. strain StRB126^25^ and 15.1% identity across QlcA from the soil metagenome^26^ and 41.5% across AiiB from *Agrobacterium tumefaciens*^27^. A phylogenetic tree of the highest similarity amino acid sequences with *aidP* from NCBI BLASTp search was also constructed (Fig. 6). *AidP* and *aidP*-homologous gene from *Planococcus* species were clustered in one clade.

## Discussion

By enrichment targeting QQ bacteria using the protocol described by Chan *et al*.^15^ at a modified lower growth temperature of 4 °C, we successfully isolated *P. versutus* L10.15^T^, a novel psychrotolerant QQ bacterium. This strain had the ability to selectively degrade AHLs with carbon side chain lengths between C_4_ and C_12_.

We infer that the QQ activity shown by strain L10.15 was achieved *via* an AHL lactonase enzyme, a class of QQ enzyme that hydrolyzes the homoserine lactone ring by cleaving its ester bond. This led us to hypothesize that *P. versutus* L10.15^T^ possesses an AHL lactonase gene similar to the *AiiA* gene, an AHL lactonase gene identified from *Bacillus* sp.^22^. RAST identified the QQ gene and revealed it to be an *N*-acyl homoserine lactonase. The BLASTp result indicated that the gene is annotated as an MBL fold hydrolase, and revealed that several other *Planococcus* species possess the MBL fold metallo-hydrolase gene with high similarity (98%) to that found in the current study. Other hits from BLASTp included genes with lower identity (85% or below) from various taxa in the order Bacillales, all of which were annotated as MBL fold metallo-hydrolases. Even though BLASTp identified several genes as AHL lactonase (Fig. 6), only AHL lactonase from *Lysinibacillus* sp. Gs50 has been characterized and confirmed in terms of its activity^21^. The QQ gene (designated as *aidP*) identified in *P. versutus* L10.15^T^ is, therefore, yet to be characterized, and could be a novel class of AHL lactonase enzyme from the metallo-β-lactamase family. This inference was further supported by the AHL lactonase MBL fold domain detected by BLASTp search. The domain was detected as a “specific hit”, indicating that the sequence has a high confidence level of the inferred function with the domain model, the *Bacillus thuringiensis* AHL lactonase (AiiA) (accession number: cd07729), with high similarity (e-value: 3.82 × 10^−78^). CDD also classified that this domain belongs to the metallo-hydrolase-like MBL fold superfamily.

The AHL lactonases from the MBL superfamily are known to have a signature zinc-binding motif HXHXDH ~ H. Crystallographic studies of AiiA^28^,^29^, AiiB^30^ and AidC^31^, have also revealed many other amino acid residues that are functionally crucial. These amino acid residues play critical functions in the catalytic mechanism. In this study, we also identify a number of functionally-important amino acid residues that were previously reported in the crystallographic studies of AiiA-type enzymes. A mutagenesis study of the active site of AiiA revealed that tyrosine (Y194) and aspartic acid (D108) residues are directly involved in the catalytic mechanism. Multiple alignment analysis of *aidP* and other AHL lactonases from the MBL superfamily revealed the presence of both amino acids (Y222 and D121) in *aidP*. The tyrosine residue provides stabilization for the substrate, while aspartic acid acts as a proton shuttle that tightens the active site, interacting with the hydroxyl leaving group of the product^30^,^32^. Asp219 (D219) of *aidP*, which is homologous with D191 of AiiA and D213 in AiiB, is important in the formation of zinc bridging. G235, which is homologous with Gly207 of AiiA, was also detected in AidP; mutation of this amino acid residue which is located in the *N-*acyl binding region will cause a significant decrease in the activity of AiiA.

In order to determine if *aidP* is conserved in the genus *Planococcus*, all available genome data of *Planococcus* sp. were subjected to RAST analysis. ‘*N*-acyl homoserine lactonase’ was found in the three closest available relatives of *P. versutus* L10.15^T^, these being the type species of *P. antarcticus, P. faecalis* and *P. kocurii*, all with high similarity (98%) to *aidP.* This indicates that these species carry a homologous *aidP* gene. Both *P. antarcticus* and *P. faecalis* were also isolated from Antarctica and, even though *P. kocurii* was not isolated from Antarctica, ANI analysis indicated that *P. faecalis* and *P. kocurii* have high OrthoANI^33^ values (98.2%) and may belong to same taxon^34^ (Supplementary Figure S5). We did not identify *N*-acyl homoserine lactonase or other QQ genes in genomes of the other *Planococcus* spp. As *aidP* and *aidP-*homologous genes have mostly been detected in the genome of *Planococcus* strains originating from Antarctica, we speculate that this may indicate the importance of QQ activity in members of *Planococcus* as a strategy for competition in harsh soil environments of the continent.

In addition to enhancing competitive ability in the Antarctic environment, the cold-active QQ enzymes of *P. versutus* L10.15^T^ could give additional advantages to this strain. Previously, *P. rifientoensis* has been reported to promote the growth of plants^35^, even though a complete genome analysis of *P. rifientoensis* identified no gene sequence coding for *N*-acyl homoserine lactonase^36^. As QQ enzymes produced by bacteria have been shown to effectively prevent the plant pathogen, *Pectobacterium* sp., from causing soft root disease on plant tubers^9^, we hypothesise a capability of *P. versutus* L10.15^T^ to promote plant growth, and a potential for it to act as a biocontrol agent in horticulture.

Using QQ as biocontrol strategy will minimize the selective pressure imposed on the targeted pathogens, and thus may reduce the development of resistance. The search for more effective QQ agents is important, as the resistance of bacteria to available antibiotics is increasing, and the development of alternative strategies against pathogens is critical^1^,^36^. It has been recognized that polar and other extreme environments are potentially important sources of novel and industrially important enzymes^37^. In this context, the discovery of psychrotolerant QQ bacteria with potential for use as biocontrol, remediation or growth promoting agents would be advantageous. In combination with the possible positive influence of nitrogen-fixing ability on plant growth, we suggest that *P. versutus* L10.15^T^ could be a beneficial bacterium to explore for applications in agriculture in cold environments.

## Materials and Methods

### Sampling location

The soil sample (upper 6 cm depth) was collected using a sterile sampling tube at the edge of an elephant seal (*Mirounga leonina*) wallow area on Lagoon Island (Ryder Bay, Adelaide Island, west of the Antarctic Peninsula; 67° 35.689′S 068° 14.495′′E). The soil sample was collected as part of a survey of bacterial communities at locations around Ryder bay (Supplementary Fig. S6).

### Bacterial isolation

For enrichment and isolation of QQ bacteria, a basal medium was used as previously described^15^. Briefly, 1 g of soil sample and 5 ml sterile medium including 100 μg C_6_-HSL were added to a sterile 50 ml plastic tube and incubated at 4 °C with shaking (150 rpm). After 1 week of incubation, 100 μl of the bacterial suspension was inoculated into fresh, sterile medium supplemented with C_6_-HSL. This step was repeated three times and finally, 100 μl of bacterial suspension was plated onto LB agar in order to obtain pure colonies.

### Bacterial strains, growth media, and culture conditions

The biosensor *C. violaceum* CV026 was used to screen for presence of AHL in the AHL inactivation assays^38^. *E. coli* DH5*α* was used as the host for cloning the 16S rDNA gene and as negative control for the AHL inactivation assay, and was grown at 37 °C in LB broth to achieve the desired OD_600_ of 1.0. Strain L10.15 was cultured at 4 °C in LB broth.

### Molecular identification and phylogenetic analysis

*P. versutus* L10.15^T^ DNA was extracted using the QIAamp DNA mini kit (Qiagen, Germany). Universal primers 27F^39^ and 1525R^40^ were used for 16S ribosomal RNA (rRNA) gene amplification. PCR products were ligated into pGEM-T following the manufacturer’s protocol (Promega, USA). DNA sequencing was performed by routine automated methods in which standard T7 forward and SP6 reverse primers were used. Phylogenetic and molecular evolutionary relationships were analysed using MEGA version 6^41^. Phylogenetic analyses of 16S rRNA were carried out using the 16S rRNA sequences of *Planococcus* type species. Alignment was performed using the MUSCLE algorithm^42^ and the phylogenies were constructed using the default settings of the neighbour-joining algorithm.

### AHL inactivation assay

To assay the ability to inactivate AHLs, synthetic AHL was dispensed into 1.5 ml sterile tubes and solvent evaporated, after which 100 μl of either *P. versutus* L10.15^T^ or *E. coli* strain DH5α cells were added to rehydrate the dried AHL (100 μM final concentration). These cell suspension mixtures were then incubated at 4 °C (except for *E. coli* DH5α at 37 °C) with shaking (220 rpm). Aliquots were withdrawn at 0 h and 24 h. The reaction was stopped by heating as described previously^37^. After cooling, the reaction mix (10 μl) was spotted onto agar overlaid with biosensor CV026 followed by overnight incubation at 28 °C. Control experiments were carried out using extraction buffer (PBS 10 mM, pH 6.5) and *E. coli* DH5α.

### Detection of AHL-degrading activity of *P. versutus* L10.15^T^ and acidification assay

The residue AHL from the assay was analysed by rapid resolution liquid chromatography (RRLC) 6400 series (Agilent, USA) using an Agilent Poroshell 120 EC-C18 column (4.6 mm × 100 mm, 2.7 μm particle size) with the elution procedure consisting of an isocratic profile of acetonitrile/water (35:65, v/v), and a constant flow rate of 0.7 mL/min, with UV detection set at 205 nm. The assay was carried out as described above, except the residue AHLs were extracted twice using an equal volume of ethyl acetate after 0 h, 24 h or 48 h of incubation, followed by evaporation to dryness. AHL extracts were reconstituted in HPLC grade acetonitrile before being subjected to RRLC analysis. For identification of AHL lactonase activity, the method of Yates *et al*.^17^ was followed. Samples withdrawn from the AHL inactivation assay were divided into two aliquots of equal volume (50 μl), and one was acidified with 100 mM HCL to pH 2.0 to induce recyclization of the lactone ring, while the other untreated aliquot was used as control. The acidified sample was incubated at 4 °C for 48 h before adjusting to pH 6.0 with MOPS buffer (0.5 M, pH 7.5).

### Whole genome sequencing and functional gene annotation

Genomic DNA of *P. versutus* L10.15^T^ was isolated using the MasterPure™ Gram-positive DNA purification kit (Epicentre Technologies) following the manufacturer’s recommended protocol. DNA quality was confirmed using a Nanodrop spectrophotometer (Thermo Scientific, USA) and Qubit^®^ 2.0 Fluorometer (Life Technologies, USA) before being constructed into a 20 kb SMRTbell template library. Pacific Biosciences (PacBio) RSII sequencing platform was used to perform whole genome sequencing using C4 chemistry in a single molecule real time (SMRT) cell. The reads were *de novo* assembled using hierarchical genome assembly process (HGAP) algorithm version 2 into a circular contig. Genome annotation was performed using the NCBI Prokaryotic Genome Annotation Pipeline (PGAP) version 2.5 and Rapid Annotation using Subsystem Technology (RAST) version 3.0^42^,^43^,^44^,^45^.

### Cloning of candidate QQ gene and confirmation of QQ activity of the candidate gene

The candidate QQ gene from *P. versutus* L10.15^T^ was amplified by PCR using the designed primer pair forward (5′-CACCATGACTGGTATTATCAAGCC) and reverse (5′- TTATTCGTAGTATCCTTCAGTCGACT) and KAPA HiFi polymerase (Kapa Biosystem, USA). PCR was performed using the following cycling parameters: 95 °C for 30 s, 62 °C for 30 s, and 72 °C for 1 min, for 35 cycles. The amplicons were purified using the AMPure XP- PCR purification system (Beckman Coulter, USA). The purified PCR product was cloned into the expression vector (pET-200) and transformed into *E. coli* BL21 Star^TM^. Next, the pET-AidP was amplified in the BL21 Star^TM^ host, and extracted using the Qiagen Plasmid Mini kit (Qiagen, Germany). pET-AidP was then transformed into expression host *E. coli* BL21 Star^TM^. The *E. coli* BL21 Star^TM^ with pET-AidP plasmid was induced by IPTG (0.4 mM) and its QQ activity was assessed as described above, except that a temperature of 16 °C was used. The AHL (C_6_-HSL with a final concentration of 2 mM) residues were subjected to liquid chromatography-mass spectrometry (LC-MS) analysis. Briefly, the samples were analyzed by LC using an Agilent Poroshell 120 EC-C18 column (4.6 mm × 100 mm, 2.7 μm particle size) with a mobile phase of acetonitrile-water (0.1% formid acid; a linear gradient [v/v] of acetonitrile from 5 to 95% over 1.5 min at a flow rate of 1.8 ml min^−1^. The separated fractions were further analyzed by electrospray ionization mass spectrometry (ESI-MS).

## Conclusions

We confirmed the *in vitro* QQ activity of *P. versutus* L10.15^T^ that produced a lactonase that degraded AHL molecules at low (4 °C) temperature, providing a new source for the isolation of these industrially important enzymes from Antarctic microbiota. *P. versutus* L10.15^T^ showed broad AHL degradation activity. Genomic analysis enabled the identification of the AHL lactonase gene, which belongs to a novel class of AHL lactonase gene. Further study is required to fully characterize this enzyme.

### Nucleotide sequence accession number

The complete genome sequence of *P. versutus* L10.15^T^ has been deposited in GenBank under the accession number CP016540.

## Additional Information

**How to cite this article:** See-Too, W. S. *et al*. *AidP*, a novel *N*-Acyl homoserine lactonase gene from Antarctic *Planococcus* sp. *Sci. Rep.* **7**, 42968; doi: 10.1038/srep42968 (2017).

**Publisher's note:** Springer Nature remains neutral with regard to jurisdictional claims in published maps and institutional affiliations.

## Supplementary Material

Supplementary Information

## Figures and Tables

**Table 1:** Genome features of *P. versutus* L10.15^T^.

Genome features	Value
**Size (bp)**	
Chromosome	3249625
Plasmid pPS15-1	70675
Plasmid pPS15-2	9775
**G + C content**	39.4%
**rRNA (5S, 16S, 23S)**	27 (9, 9, 9)
**tRNA genes**	71
**Number of coding sequences (CDS)**	3153

**Table 2:** List of domain hits of NCBI Conserved Domain Database for *AidP* with E-value threshold of 0.01.

Name	Accession	Description	Interval	E-value
AHL lactonase MBL-fold	cd07729	*N*-acyl-L-homoserine lactone hydrolase, MBL-fold metallo-hydrolase domain; These enzymes belong to the MBL-fold metallo-hydrolase superfamily.	10–266	3.82 × 10^−78^
Lactamase B	pfam00753	Metallo-beta-lactamase superfamily;	47–265	6.66 × 10^−15^
Lactamase B	smart00849	Metallo-beta-lactamase superfamily; Apart from the beta-lactamases a number of other proteins contain this domain. These proteins include thiolesterases, members of the glyoxalase II family, that catalyse the hydrolysis of S-D-lactoyl-glutathione to form glutathione and D-lactic acid and a competence protein that is essential for natural transformation in *Neisseria gonorrhoeae* and could be a transporter involved in DNA uptake. Except for the competence protein these proteins bind two zinc ions per molecule as cofactor.	50–265	1.44 × 10^−14^
COG1237	COG1237	Metal-dependent hydrolase, beta-lactamase superfamily II [General function prediction only];	52–188	3.87 × 10^−9^
GloB	COG0491	Glyoxylase or a related metal-dependent hydrolase, beta-lactamase superfamily II [General function prediction only]	40–266	5.32 × 10^−14^
arCOG00543	TIGR03675	arCOG00543 universal archaeal KH-domain/beta-lactamase-domain protein; This family of proteins is universal in the archaea and consists of an N-terminal type-1 KH-domain (pfam00013) a central beta-lactamase-domain (pfam00753) with a C-terminal motif associated with RNA metabolism (pfam07521). KH-domains are associated with RNA-binding, so taken together, this protein is a likely metal-dependent RNAase. This family was defined as arCOG01782.	52–129	5.26 × 10^−5^
