# Supplementary material for: AidP, a novel N-Acyl homoserine lactonase gene from Antarctic Planococcus sp
Source: Sci Rep. 2017 Feb 22;7:42968. doi: 10.1038/srep42968 (PMC5320481; doi:10.1038/srep42968)
Supplement: Supplementary Information [file srep42968-s1.pdf]

***AidP,* a novel *N*-Acyl Homoserine Lactonase gene from Antarctic *Planococcus* sp.**

Wah Seng See-Too[1,2], Robson Ee[1], Yan-Lue Lim[1], Peter Convey [2,3], David A. Pearce[2,3,4], Wai-Fong Yin[1], Kok-Gan Chan*[1,5]

[1] Division of Genetics and Molecular Biology, Institute of Biological Sciences, Faculty of Science University of Malaya, 50603 Kuala Lumpur, Malaysia

[2] National Antarctic Research Centre (NARC), Institute of Postgraduate Studies, University of Malaya, 50603 Kuala Lumpur, Malaysia

[3]British Antarctic Survey, NERC, High Cross, Madingley Road, Cambridge CB3 OET, UK

[4]Faculty of Health and Life Sciences, University of Northumbria, Newcastle Upon Tyne NE1 8ST, UK

[5]UM Omics Centre, University of Malaya, Kuala Lumpur, Malaysia

*Corresponding author: Institute of Biological Sciences (Division of Genetics and Molecular Biology), Faculty of Science, University of Malaya, 50603 Malaysia. Tel: +603-79675162.

Email: kokgan@um.edu.my

Supplementary Data:

Supplementary Table 1. Top 30 BLASTN comparison hits of strain L10.15 chromosomal contig (3.2Mb) against NCBI non-redundant nucleotide database.

Description
Planococcus sp. L10.15, complete genome
Planococcus kocurii strain ATCC 43650, complete genome
Planococcus sp. PAMC 21323, complete genome
Planococcus halocryophilus strain DSM 24743, complete genome
Planococcus donghaensis strain DSM 22276, complete genome
Planococcus antarcticus DSM 14505, complete genome
Planococcus maritimus strain DSM 17275, complete genome
Planococcus rifietoensis strain M8, complete genome
Planococcus plakortidis strain DSM 23997, complete genome
Planococcus donghaensis strain DSM 22276 plasmid pPD76, complete sequence
Bacillus sp. FJAT-22090 genome
Rummeliibacillus stabekisii strain PP9, complete genome
Lysinibacillus varians strain GY32, complete genome
Lysinibacillus fusiformis strain RB-21, complete genome
Lysinibacillus sphaericus strain 2362, complete genome
Lysinibacillus sphaericus III(3)7, complete genome
Lysinibacillus sphaericus strain OT4b.25, complete genome
Lysinibacillus sphaericus C3-41, complete genome
Lysinibacillus sp. 13S34_air genome assembly PRJEB5506_assembly_1, scaffold CONTIG000001
Solibacillus silvestris strain DSM 12223, complete genome
Solibacillus silvestris StLB046 DNA, complete genome
Kurthia sp. 11kri321, complete genome
Sporosarcina psychrophila strain DSM 6497, complete genome
Bacillus sp. OxB-1 DNA, complete genome
Jeotgalibacillus malaysiensis strain D5, complete genome
Bacillus sp. 1NLA3E, complete genome
Bacillus oceanisediminis 2691, complete genome
Bacillus sp. X1(2014), complete genome
Bacillus simplex strain SH-B26, complete genome
Bacillus muralis strain G25-68, complete genome
Bacillus sp. Alg07, complete genome

Supplementary Table 2. Top 30 BLASTN comparison hits of pPS15-1 plasmid sequence (70.7 kb) against NCBI non-redundant nucleotide database.

Description
Planococcus sp. L10.15 plasmid pPS15-1, complete sequence
Planococcus sp. PAMC 21323, complete genome
Planococcus kocurii strain ATCC 43650, complete genome
Planococcus donghaensis strain DSM 22276, complete genome
Planococcus halocryophilus strain DSM 24743, complete genome
Planococcus antarcticus DSM 14505, complete genome
Planococcus sp. L10.15, complete genome
Planococcus maritimus strain DSM 17275, complete genome
Staphylococcus aureus strain 1128105 plasmid p1128105, partial sequence
Staphylococcus aureus strain 1 plasmid pSA8589, complete sequence
Proteus vulgaris strain PV-01 insertion sequence IS26, complete sequence; and rRNA methylase (cfr) gene, complete cds
Bacillus sp. OxB-1 DNA, complete genome
Planococcus plakortidis strain DSM 23997, complete genome
Sporosarcina psychrophila strain DSM 6497, complete genome
Bacillus amyloliquefaciens strain S499, complete genome
Bacillus amyloliquefaciens IT-45, complete genome
Lactococcus lactis subsp. lactis bv. diacetylactis plasmid pVF22, complete sequence
Lactococcus lactis bv. diacetylactis SpoVJ-like protein gene, partial cds; putative resolvase, restriction enzyme, putative trasnposase, 5-methylcytosine methyltransferase, and putative transposase genes, complete cds; and unknown ge
Streptococcus equinus strain 1357 hypothetical protein genes, complete cds; insertion sequence IS1216-like, complete sequence; and hypothetical protein, TetS/M (tetS/M), potassium transport system protein, putative resolvase, and [illegible]
Lactococcus lactis subsp. lactis strain S0, complete genome
Lactococcus lactis subsp. lactis CV56 plasmid pCV56B, complete sequence
Lactococcus lactis subsp. lactis CV56 plasmid pCV56A, complete sequence
Lactococcus lactis subsp. cremoris NZ9000, complete genome
Lactococcus lactis subsp. cremoris MG1363, complete genome
Lactococcus lactis subsp. cremoris Lin (lin) and putative transposase gene, complete cds; putative transposase gene, partial cds; and unknown gene
Lactococcus lactis subsp. lactis NCDO 2118 plasmid pNCDO2118, complete sequence
Lactococcus lactis subsp. lactis KF147 plasmid pKF147A, complete genome
Arsenophonus nasoniae whole genome shotgun assembly, contig scaffold01110
Paenibacillus polymyxa strain YC0573, complete genome
Paenibacillus peoriae strain HS311, complete genome

Supplementary Table 3. Top 30 BLASTN comparison hits of pPS15-2 plasmid sequence (9.8 kb) against NCBI non-redundant nucleotide database.

Description
Planococcus sp. L10.15 plasmid pPS15-2, complete sequence
Planococcus sp. L10.15, complete genome
Planococcus citreus plasmid pNM11, complete sequence
Planococcus antarcticus DSM 14505 plasmid pPA05-1, complete sequence
Planococcus kocurii strain ATCC 43650 plasmid, complete sequence
Planococcus citreus plasmid pNM8, complete sequence
Planococcus sp. ZOYM plasmid pPCZ2, complete sequence
Planococcus halocryophilus strain DSM 24743, complete genome
Staphylococcus sciuri pSCFS1 plasmid, complete sequence
Planococcus antarcticus DSM 14505 plasmid pPA05-2, complete sequence
Planococcus sp. PAMC 21323 plasmid pPla, complete sequence
Rummeliibacillus stabekisii strain PP9 plasmid pPP9, complete sequence
Planococcus antarcticus DSM 14505, complete genome
Paenibacillus sp. IHBB 10380, complete genome
Anoxybacillus gonensis strain G2, complete genome
Exiguobacterium sp. AT1b, complete genome
Planococcus kocurii strain ATCC 43650, complete genome
Geobacillus stearothermophilus 10, complete genome
Geobacillus thermoglucosidasius C56-YS93, complete genome
Geobacillus sp. Y412MC52, complete genome
Geobacillus sp. Y412MC61, complete genome
Parageobacillus thermoglucosidans strain TM242, complete genome
Parageobacillus thermoglucosidans strain NCIMB 11955, complete genome
Geobacillus thermoglucosidasius strain DSM 2542, complete genome
Geobacillus sp. C56-T3, complete genome
Paenibacillus sp. DCY84, complete genome
Geobacillus sp. Y4.1MC1, complete genome
Geobacillus sp. LC300, complete genome
Paenibacillaceae bacterium GAS479 genome assembly, chromosome: I
Planococcus plakortidis strain DSM 23997, complete genome

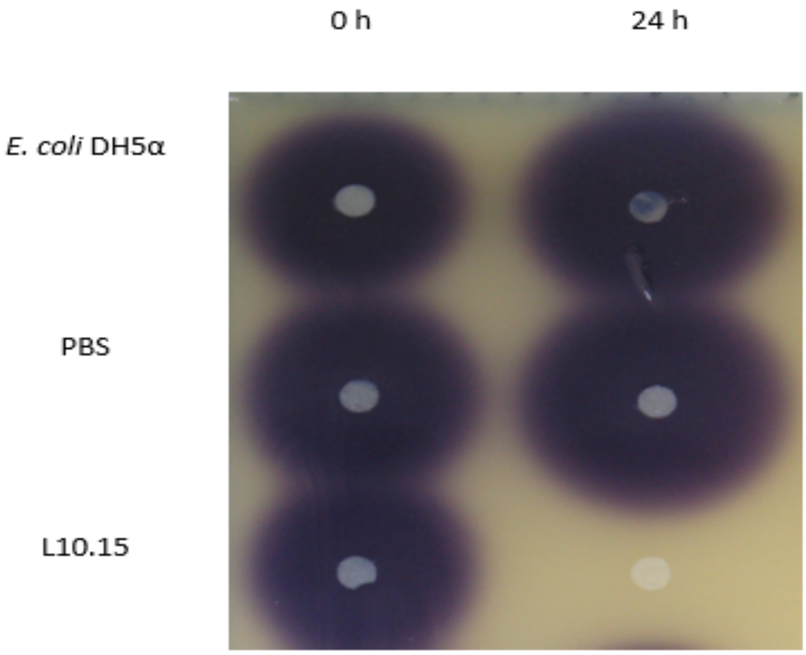

Supplementary Figure S1: The synthetic AHL (C_6-HSL) was incubated with the resting cell of L10.15 in 4 °C for 24 h. C_6-HSL was completely degraded after 24 h. The first 2 lanes are negative control (*E.coli* DH5α and PBS), the left row is the AHL harvested at 0 h, and the right row is AHL harvested after 24 h incubation.

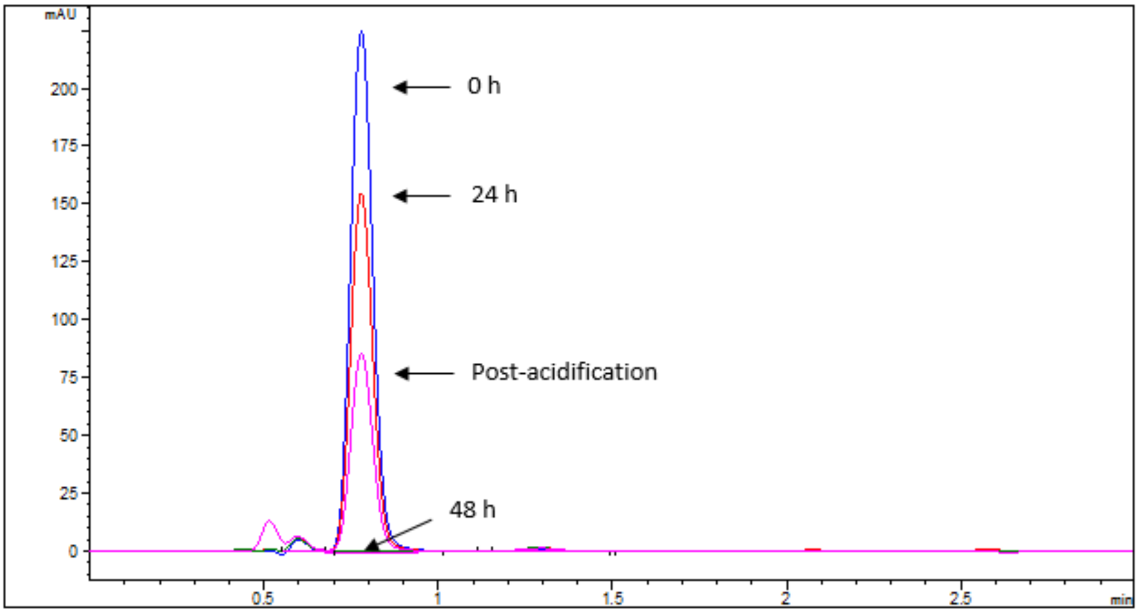

Supplementary Figure S2: Acidification assay of *Planococcus* sp. strain L10.15 after AHL inactivation assay. A significant proportion of AHL activity was restored, indicating re-lactonasation of AHL.

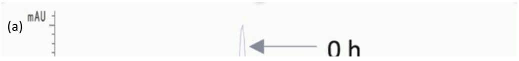

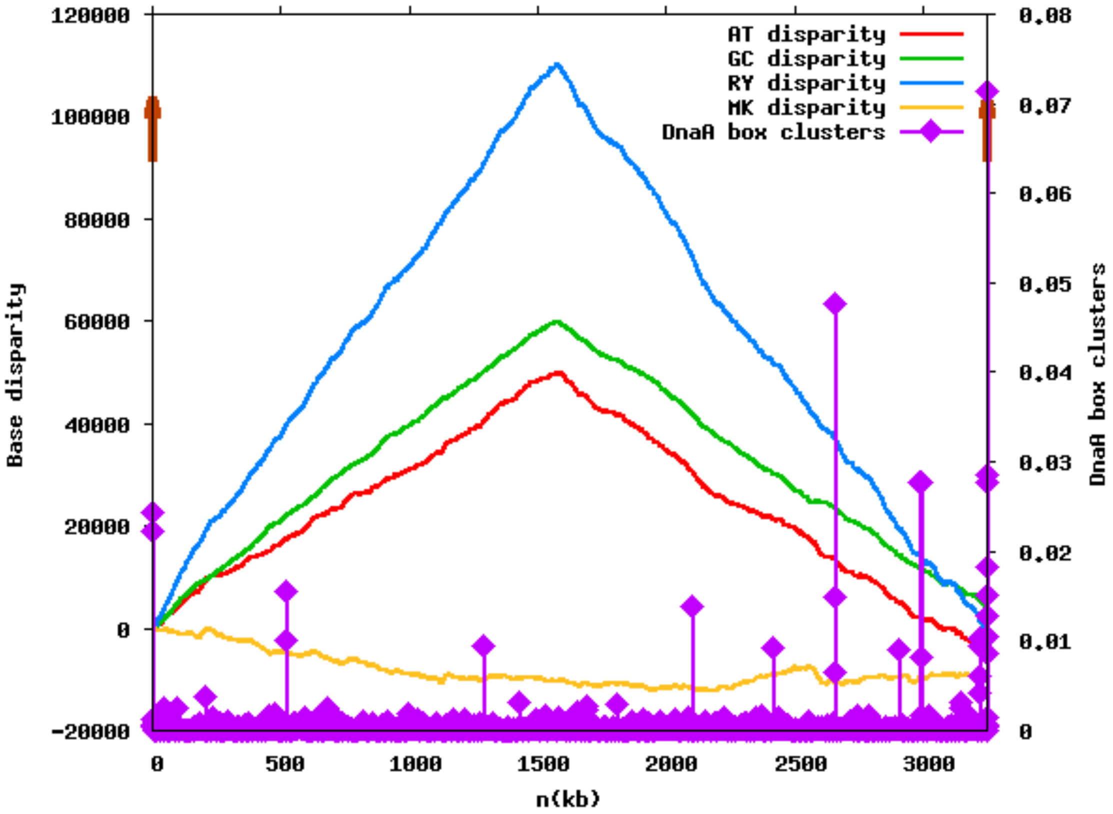

Supplementary Figure S3: Z-curve plot (with AT, GC, RY and MK disparity curves) for the chromosomal contig sequence of *P. versutus* L10.15^{T} . The minimum point of the inverted V shaped plot of both AT and GC disparity curve correspond to the location of the predicted *oriC* region whereas the maximum point indicated the location of the terminus of replication. The predicted origin and the terminus of replication of the chromosome can be observed to divide the chromosome into two equidistant parts.

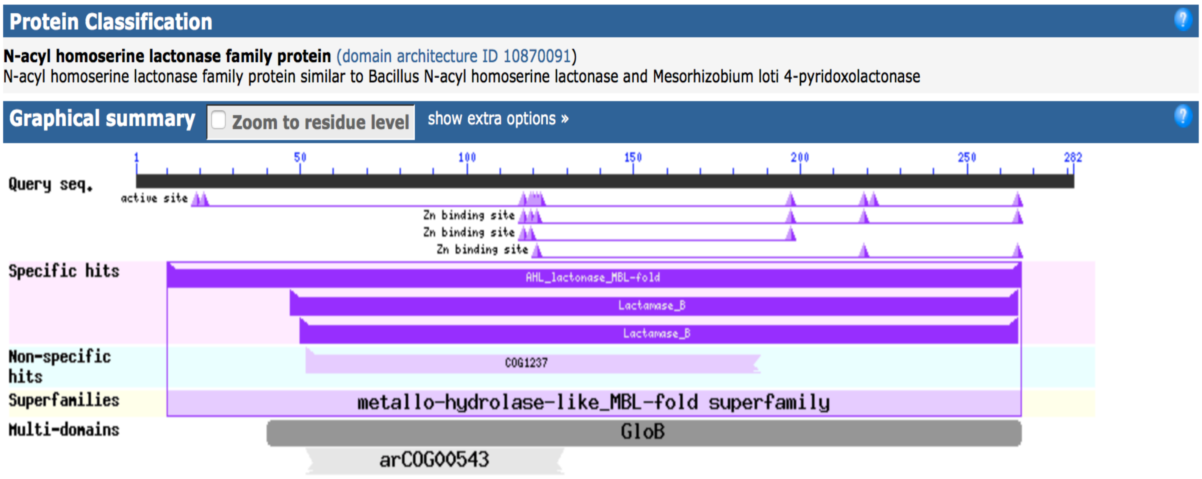

Supplementary Figure S4. Conserved domain detected by NCBI BLASTp search.

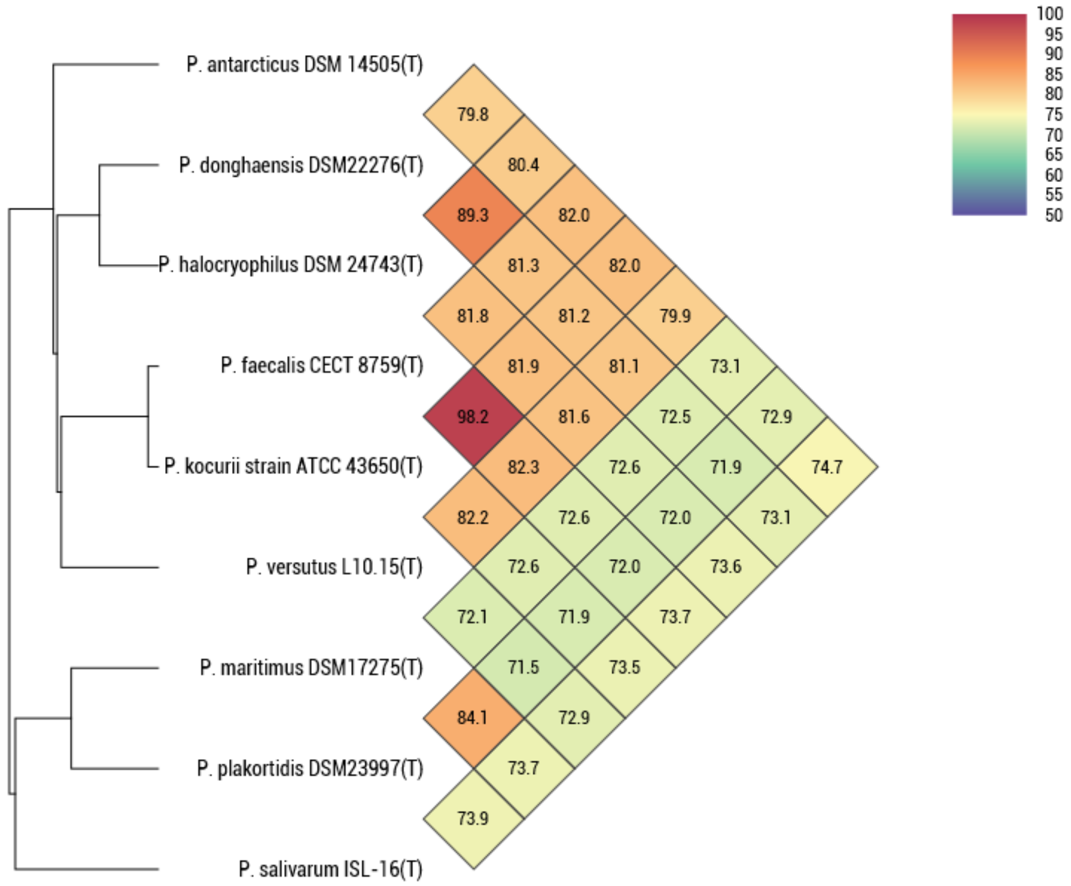

Supplementary Figure S5: OrthoANI analysis of *P.versutus* L10.15^T and other type strains of *Planococcus* sp.

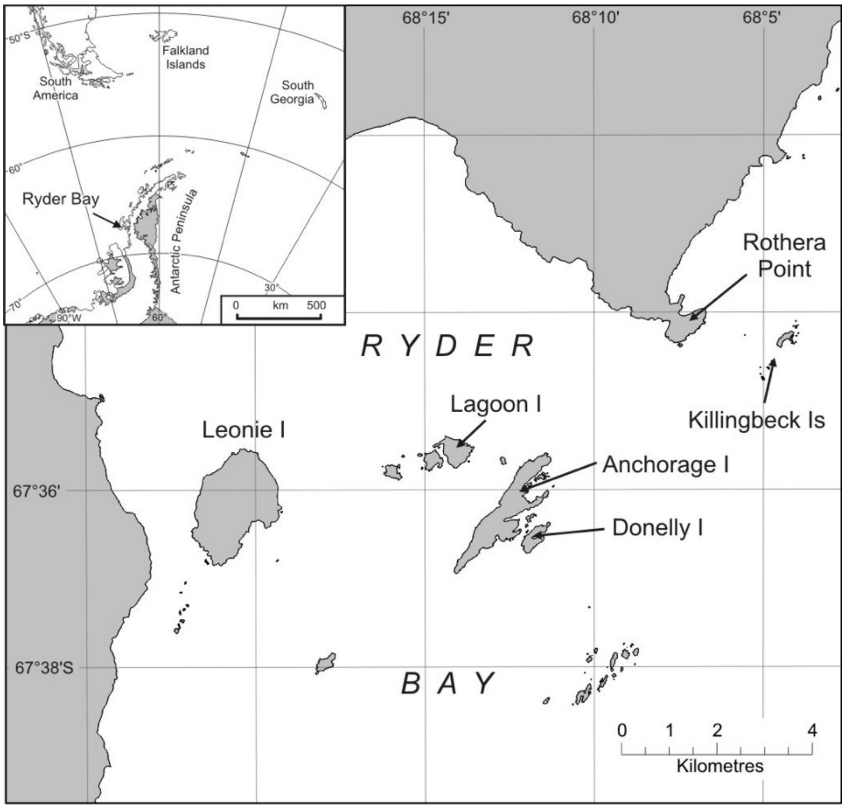

Supplementary Figure S6: The location of Lagoon Island in Ryder Bay, south-eastern Adelaide Island, off the west coast of the Antarctic Peninsula. The map was generated using ArcGIS (version 9.3) from data contained in the Antarctic Digital Database (http://www.add.scar.org/home/add7), and finished in Corel Draw (version 14).
